# SFTSV NSs degrades SAFA via autophagy to suppress SAFA-dependent antiviral response

**DOI:** 10.1371/journal.ppat.1013201

**Published:** 2025-06-03

**Authors:** Tian-mei Yu, Ze-min Li, Wen-kang Zhang, Bang Li, Qiao Liu, Chuan-min Zhou, Xue-jie Yu

**Affiliations:** 1 State Key Laboratory of Virology, School of Public Health, Wuhan University, Wuhan, China; 2 Gastrointestinal Disease Diagnosis and Treatment Center, The First Hospital of Hebei Medical University, Shijiazhuang, China; Universite Laval Faculte de medecine, CANADA

## Abstract

Severe fever with thrombocytopenia syndrome virus (SFTSV), a tick-borne bunyavirus, causes an emerging viral hemorrhagic fever with a high mortality rate. SFTSV nonstructural protein S (NSs) is a virulence factor that sequesters antiviral proteins into autophagic vesicles for degradation to escape host immune response. SAFA (Nuclear scaffold attachment factor A), an RNA sensor, recognizes viral RNA and is retained in the cytoplasm upon RNA virus SFTSV infection and then activates innate immunity. It is unclear whether NSs mediates the escape of SAFA-mediated antiviral response. Here we showed that SFTSV NSs can inhibit SAFA-dependent antiviral response via autophagy. We used SAFA-NLS (the nuclear localization signal) mutant to transfect SAFA knocked-out MEF cells and found that the cytoplasmic SAFA promoted innate immune response to poly(I:C) stimulating. Importantly, NSs interacted with the AAA+ domain of SAFA and retained SAFA in the cytoplasm thereby suppressing SAFA-mediated antiviral response. Mechanistically, SFTSV NSs degraded cytoplasmic SAFA via SQSTM1/p62-dependent autophagy and sequestered SAFA into autophagic vesicles for degradation through promoting the interaction between SAFA and LC3. In conclusion, our results indicate a novel mechanism of SFTSV NSs to escape host antiviral immune response by recruiting SAFA into autophagic flux for degradation.

## Author summary

Severe fever with thrombocytopenia syndrome virus (SFTSV) is a tick-borne bunyavirus which causes an emerging viral hemorrhagic fever with a high mortality rate. The nonstructural protein (NSs) of SFTSV is an important viral virulence factor that inhibits host antiviral immune responses. SAFA (Nuclear scaffold attachment factor A) is a nuclear RNA sensor which senses viral RNA and nucleoprotein (NP) in the cytoplasm to initiate innate immunity against RNA virus SFTSV infection. Here we found that SFTSV NSs retained SAFA in the cytoplasm through interaction with its AAA+ domain and sequestered SAFA into autophagic vesicles for degradation by promoting the interaction between SAFA and LC3 via SQSTM1/p62 autophagy. We also found that a SAFA-NLS mutant that lose the ability to enter nuclear and therefore is retained in cytoplasm still activated antiviral immunity. Our findings reveal a novel mechanism of SFTSV NSs to escape host antiviral immune response by recruiting SAFA into autophagic flux for degradation.

## Introduction

Severe fever with thrombocytopenia syndrome virus (SFTSV), a tick-borne bunyavirus belonging to genus *Bandavirus* of the family Phenuiviridae and order Bunyavirales, was first discovered in China in 2009 and had been reported in East and Southeast Asian countries [1–6]. SFTSV is mainly transmitted by ticks and occasionally through body fluids of patients or infected animals [7,8]. SFTSV infection causes a severe viral hemorrhagic fever termed SFTS with clinical manifestations of fever, thrombocytopenia, leukopenia, gastrointestinal symptoms, and the potential development of multiple organ dysfunction, with a mortality rate reaching up to 30% [2,9]. Currently, there are no effective therapeutics or vaccines available for SFTS prevention and treatment. In view of the threat posed by SFTSV to human health, the World Health Organization has listed SFTSV in the Blueprint Priority Diseases in 2024 [10].

SFTSV is an enveloped, negative-sense single-stranded RNA virus and its genome consists of three segments: the large (L), the medium (M), and the small (S) RNA segments. The L segment encodes RNA-dependent RNA polymerase (RdRp) mediating the transcription and replication of viral genomes. The M segment encodes glycoprotein (GP) which modified by host proteases to form N-terminal glycoprotein (Gn) and C-terminal glycoprotein (Gc), mediating virion assembly and entry to target cells. The S segment is ambisense that encodes the nucleoprotein (NP) promoting formation of SFTSV ribonucleoprotein complexes (RNPs), and the nonstructural proteins S (NSs), a virulence factor to inhibit host antiviral immune responses [1].

The innate immune response is the first line against invading pathogens. Host cells recognize pathogen-associated molecular patterns (PAMPs) through different pattern recognition receptors (PRRs) to induce innate immune responses and resist viral invasion [11]. SFTSV could evade the host immune response through a variety of strategies with NSs forming viroplasm-like structures (VLSs) to sequester multiple interferon-related proteins including TBK1 (TANK binding kinase 1), IKKε (inhibitor of nuclear factor kappaB kinase subunit epsilon), RIG-I (retinoic acid-inducible gene I), TRIM25 (E3 ubiquitin ligase), STAT1 and STAT2 (signal transducer and activator of transcription 1 and 2), IRF3 and IRF7 (interferon regulatory factor 3 and 7) and NP stimulating autophagy to degrade cGAS (cyclic GMP-AMP synthase) and MAVS (mitochondrial antiviral-signaling protein) [12–17]. Recently, we demonstrated that SFTSV NSs can induce complete autophagic flux and hijack antiviral proteins such as TBK1 and TRIM25 into autophagic vesicles for degradation and escaping host immune responses [18].

Nuclear matrix protein-nuclear scaffold attachment factor A (SAFA), also known as heterogeneous ribonucleoprotein U, is the largest member of the hnRNP family. SAFA was originally identified as a nuclear DNA-binding protein with a high affinity for nuclear matrix or scaffold-attached DNA elements [19]. Recently, SAFA was identified as a nuclear RNA sensor [20]. Upon recognition of viral double-stranded RNA in nucleus, SAFA oligomerizes and interacts with SMARCA5 and TOP1 to activate the enhancers of antiviral gene and super enhancers, thereby regulating the antiviral response [20]. Our previous study showed that SAFA functioned as an RNA PRR which recognized RNA virus SFTSV NP and RNA in the cytoplasm, and then activated STING-TBK1-dependent signaling cascades to induce anti-SFTSV response [21]. Considering the ability of NSs to inhibit antiviral innate immunity, it is speculated that SFTSV NSs may limit the SAFA-mediated antiviral response to achieve immune escape. In this study, we aim to explore the connections between the NSs and SAFA, which will further understand the interactions between SAFA and SFTSV and help explore new mechanisms by which SFTSV NSs inhibit antiviral immune responses.

## Results

### SAFA-NLS activates the anti-viral immune response and inhibits SFTSV replication

In normal cells, SAFA is located purely in the nucleus and does not shuttle between the nucleus and cytoplasm [22,23]. However, upon RNA virus SFTSV infection, SAFA is retained in the cytoplasm to activate type I IFN signaling. To explore the function of SAFA in the cytoplasm, we utilized a SAFA-NLS mutant (E240A-R240A) which lost the ability to enter the nucleus and always retained in the cytoplasm [21]. Western blot analysis confirmed the absence of SAFA protein expression in the SAFA^-/-^ MEF cells (S1A Fig). We transfected the SAFA knocked-out MEF cells with the Flag-SAFA-NLS-mutant plasmid. Western blot and Confocal microscopy showed that the SAFA-NLS mutant retained in the cytoplasm (S1B and S1C Fig). RNA binding protein immunoprecipitation (RIP) assay showed that SAFA-NLS mutant can pull down viral RNAs of both SFTSV S and M segment under SFTSV infection (S1D Fig).

**Fig 1:**
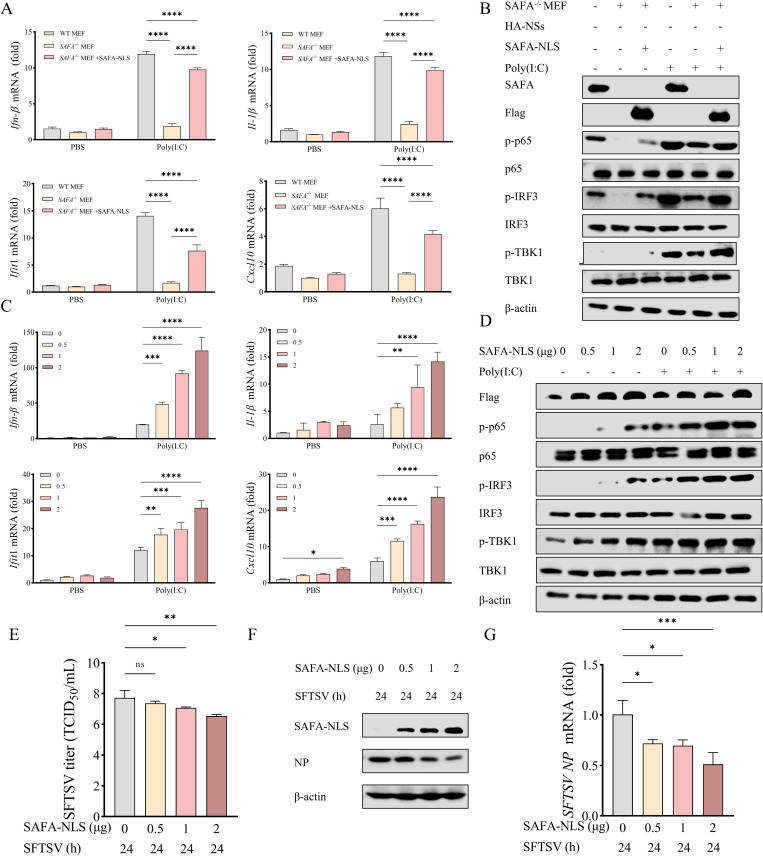
SAFA-NLS activates the anti-viral immune response and inhibits SFTSV replication. (A-B) WT MEF and *SAFA*^-/-^ MEF cells were transfected with Flag-SAFA-NLS plasmid or empty vector (pCDNA3.1) for 24 h, and then stimulated with poly(I:C) (1μg/μL) or PBS as control for 24 h, the mRNA levels of *Ifn-*β, *Il*1*-*β, *Ifit*1*, Cxcl*10 were determined with RT-qPCR (A). The phosphorylation levels of TBK1, IRF3 and p65 were detected with western blot using anti-p-TBK1, anti-p-IRF3 and anti-p-p65 antibodies (B). (C-D) *SAFA*^-/-^ MEF cells were transfected with increasing amounts of Flag-SAFA-NLS plasmid or empty vector pCDNA3.1 for 24 h, and then stimulated with poly(I:C) or PBS control for 24 h. The mRNA levels of *Ifn-*β, *Il*1*-*β, *Ifit*1*, Cxcl*10 and the phosphorylation levels of TBK1, IRF3 and p65 were determined with RT-qPCR **(C)** and western blot, respectively (D). (E-G) *SAFA*^-/-^ MEF cells were transfected with increasing amounts of Flag-SAFA-NLS plasmids for 24 h, and then infected with SFTSV (MOI = 5) for 24 h. SFTSV titers **(E)** in the culture supernatants were measured using the TCID_50_ assay. SFTSV NP protein **(F)** and mRNA **(G)** levels in cells were detected with western blot using anti-NP antibodies and RT-qPCR.

RT-qPCR and western blot were utilized to explore the ability of cytoplasmic SAFA to activate innate immunity. The results showed that the SAFA-NLS mutant could rescue the reduction of *Ifn-*β, *Il*1*-*β, *Ifit*1*, Cxcl*10 mRNA and the decreased phosphorylation of TBK1, IRF3 and p65 caused by SAFA knockdown (Fig 1A and 1B). Furthermore, the mRNA levels of *Ifn-*β, *Il*1*-*β, *Ifit*1*, Cxcl*10 and the phosphorylation level of TBK1, IRF3 and p65 were dependent on the overexpressing level of SAFA-NLS mutant in *SAFA*^*-/-*^ MEF cells (Fig 1C and 1D). To examine the effects of SAFA-NLS mutant on SFTSV infection, the viral titers in the supernatants of cells overexpressing SAFA-NLS was titrated with TCID_50_. The result showed that the titer of SFTSV in the supernatant of *SAFA*^-/-^ MEF that overexpressed SAFA-NLS mutant was significantly decreased in a dose dependent manner (Fig 1E). In addition, SFTSV NP protein and mRNA levels were downregulated as the quantity of SAFA-NLS plasmids increased (Fig 1F and 1G). These results suggest that SAFA-NLS mutant as cytoplasmic SAFA promoted antiviral immune response and had the function of inhibiting SFTSV replication.

### SFTSV NSs interacts with SAFA and mediates its translocation

**Fig 2:**
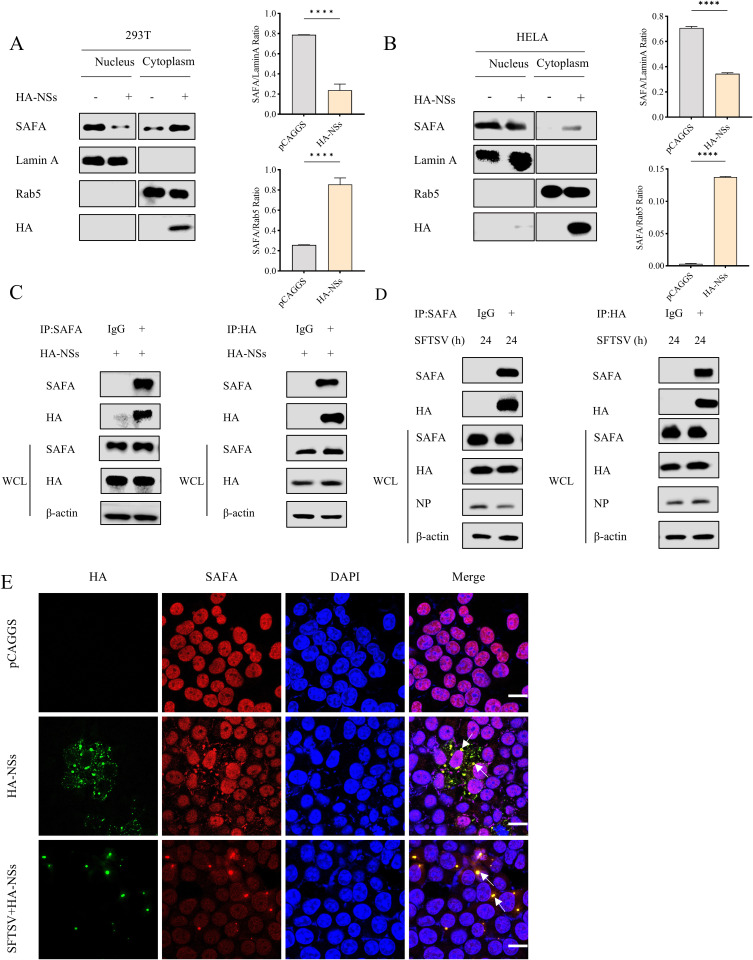
SFTSV NSs interacts with SAFA and mediates the translocation of SAFA. (A-B) 293T **(A)** and Hela **(B)** cells were transfected with vector pCAGGS or HA-NSs plasmid. Cells were then collected at 24 h for nuclear and cytosolic fractionations. Lamin A and Rab5 were nuclear and cytoplasmic index proteins, respectively. (C-D) 293T cells were transfected with HA-NSs for 24 h **(C) or** 293T cells were infected with SFTSV for 2 h and then transfected with HA-NSs for 24 h for simulating the infection status (D). The interaction between endogenous SAFA and SAFA-NLS and NSs was analyzed using co-IP assay with IgG as control. (E) 293T cells were transfected with pCAGGS or HA-NSs for 24 h and then with or without SFTSV (MOI = 5) infection for 24 h. HA (green), SAFA (red), and DAPI (blue) were analyzed with confocal microscopy. Nuclei were stained with DAPI (blue). Scale bar: 20 μm.

To explore the effect of SFTSV NSs on SAFA, HA-NSs plasmid were transfected into 293T (HEK293T) and HeLa cells. As expected, western blot showed that the SAFA level in the cytoplasm increased in the SFTSV NSs overexpression group (Fig 2A and 2B). To further explore the interaction between NSs and SAFA, we transfected 293T cells with HA-NSs. Co-immunoprecipitation (co-IP) assays showed that NSs can pull down endogenous SAFA and vice versa (Fig 2C). Consistently, endogenous SAFA was pulled down by SFTSV NSs in SFTSV infected cells (Fig 2D). Confocal microscopy was utilized to examine the colocalization of NSs and SAFA. The results showed that SFTSV NSs highly colocalized with endogenous SAFA (Fig 2E). The Flag-SAFA-NLS mutant and HA-NSs plasmids were co-transfected into 293T cells, and the results showed that cytoplasmic SAFA can directly interact with NSs (S2 Fig). The above results indicate that the SFTSV NSs can interact with SAFA and mediate its translocation from nucleus to cytoplasm.

### SFTSV NSs interacts with the AAA+ domain of SAFA

**Fig 3:**
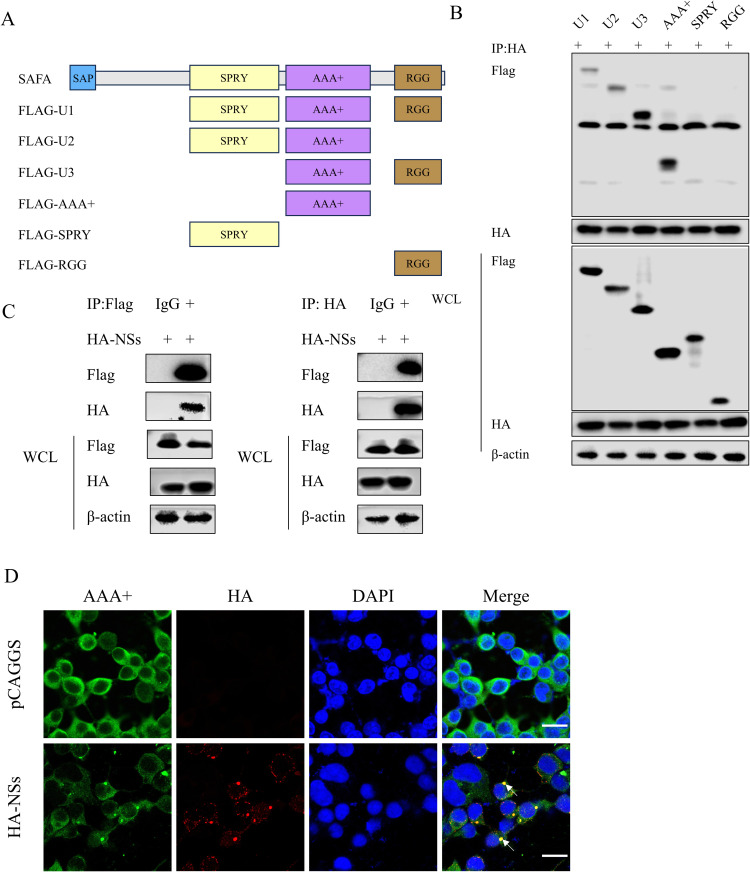
SFTSV NSs interacts with the AAA+ domain of SAFA. (A) The schematic diagram of SAFA truncation mutants. (B) 293T cells were co-transfected with HA-NSs and Flag-U1, Flag-U2, Flag-U3, Flag-AAA + , Flag-SPRY, or Flag-RGG, the interaction between truncations and HA-NSs was analyzed with co-IP assays. (C-D) 293T cells were co-transfected with Flag-AAA+ with pCAGGS or HA-NSs for 24 h and were analyzed with co-IP **(C)** and confocal assays (D). HA (red), Flag (green), and DAPI (blue) were analyzed with confocal microscopy. Nuclei were stained with DAPI (blue). Scale bar: 20 μm.

To determine the interaction site of SAFA with NSs, we constructed six SAFA truncations based on its functional domains (Fig 3A). The full-length protein, spanning 825 amino acids, is divided into four domains: the DNA-binding SAP domain, the SPRY protein–protein interaction domain, the AAA+ oligomerization domain and the RNA-binding domain (RGG) [24]. Each SAFA truncations were transfected into 293T cells together with HA-NSs. Co-IP assays showed that the NSs could pull down truncations containing AAA+ domain, indicating that the AAA+ domain of SAFA was the site to interact with the NSs (Fig 3B). Further co-IP assays and confocal microscopy confirmed the interaction between the AAA+ domain of SAFA and HA-NSs (Fig 3C and 3D).

### The SFTSV NSs inhibits the SAFA-NLS mediated innate immune response

**Fig 4:**
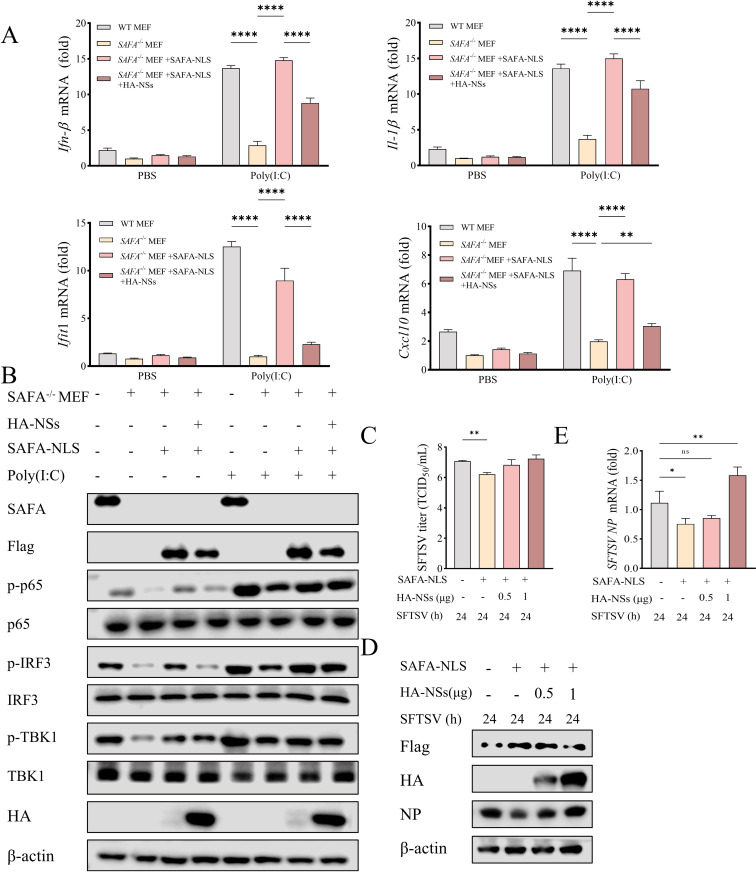
The SFTSV NSs inhibits the SAFA-NLS mediated innate immune response. (A-B) WT MEF and *SAFA*^-/-^ MEF cells were co-transfected with the pCDNA 3.1 or Flag-SAFA-NLS plasmid with pCAGGS or HA-NSs for 24 h, and then stimulated with PBS or poly(I:C) for 24 h, the mRNA levels of *Ifn-*β, *Il*1*-*β, *Ifit*1*, Cxcl*10 were detected with RT-qPCR (A). The levels of phosphorylation of TBK1, IRF3 and p65 were detected with western blot (B). (C-E) *SAFA*^*-/-*^ MEF cells were transfected with the Flag-SAFA-NLS along or with HA-NSs for 24 h, and then infected with SFTSV (MOI = 5) for 24 h. SFTSV titers **(C)** in the culture supernatants were measured using the TCID_50_. SFTSV NP protein **(D)** and mRNA **(E)** levels in cells were detected with western blot and RT-qPCR.

To investigate whether SFTSV NSs inhibits SAFA-mediated antiviral immune response in cytoplasm, we co-transfected SAFA-NLS mutant and HA-NSs plasmids into *SAFA*^-/-^MEF cells. RT-qPCR showed that NSs overexpression inhibited poly(I:C)-stimulated SAFA-NLS-induced transcription of *Ifn-*β, *Il*1*-*β, *Ifit*1*, Cxcl*10 (Fig 4A). NSs inhibited the increases of the protein levels of p-TBK1*,* p-IRF3 and p-p65 induced by SAFA-NLS (Fig 4B). The above results indicate that NSs can inhibit the innate immune response mediated by cytoplasmic SAFA. Furthermore, TCID_50_, western blot and RT-qPCR showed that NSs overexpression abolished the inhibitory effect of SAFA on SFTSV replication and the mRNA and protein levels of SFTSV NP (Fig 4C-4E). These results demonstrate that SFTSV NSs can mediate immune escape through inhibiting cytoplasmic SAFA signaling pathway, which further affects its proliferation.

### SFTSV NSs degrades SAFA through autophagy

**Fig 5:**
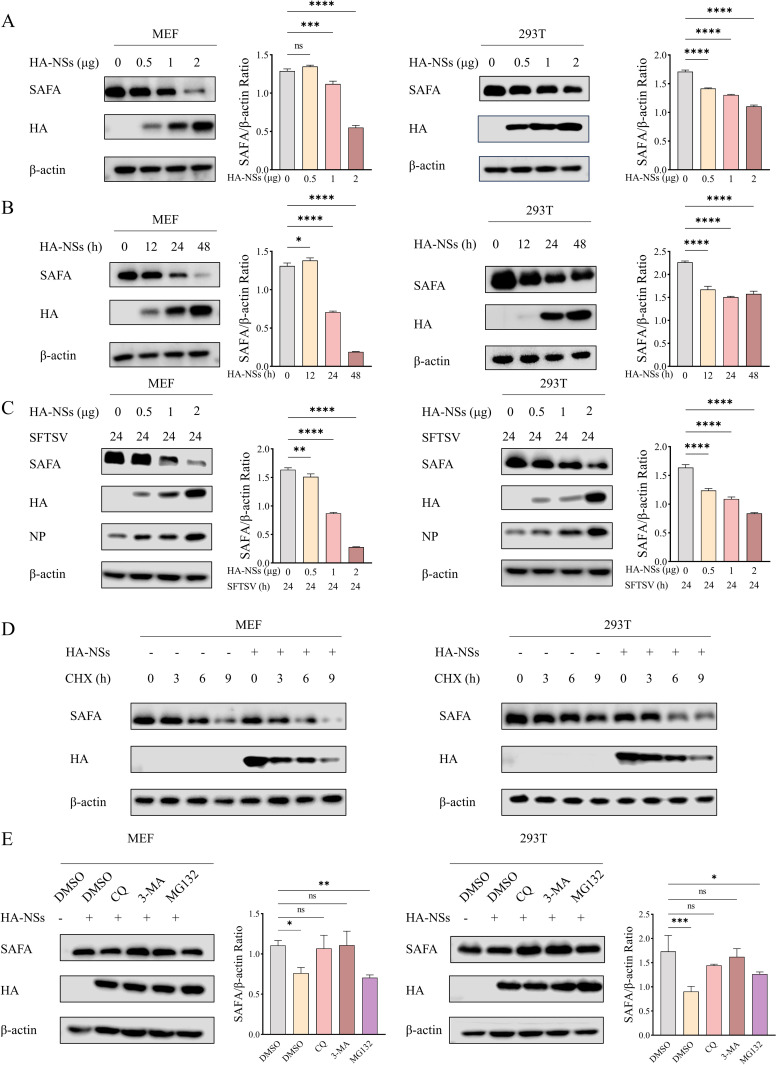
SFTSV NSs degrades SAFA through autophagy. (A) MEF and 293T cells were transfected with the increasing amount of HA-NSs plasmid for 24 h. (B) MEF and 293T cells transfected with HA-NSs plasmid were cultivated and harvested at different time points. (C) MEF and 293T cells were infected with SFTSV (MOI = 5) for 2 h and then transfected with HA-NSs plasmid for 24 h. (D) MEF and 293T cells were transfected with HA-NSs plasmid or pCAGGS for 24 h, the resultant cells were treated with cycloheximide (25 μg/ml) for 0, 3, 6, 9 h. (E) MEF cells were transfected with HA-NSs for 24 h, and then the cells were treated with DMSO, CQ (5 μM), 3-MA (1 mM), and MG132 (5 μM). The protein levels of SAFA were detected with western blot in all experiments.

To clarify the mechanism of the NSs inhibits SAFA-mediated innate immune response, we first investigated the effects of NSs on SAFA. MEF and 293T cells were transfected with HA-NSs plasmid. Western blot showed that overexpression of HA-NSs inhibited the protein level of endogenous SAFA in a dose-dependent manner, while HA-NSs did not affect the mRNA level of SAFA (Figs 5A and S3A). In addition, HA-NSs decreased endogenous SAFA protein level in a time-dependent manner (Fig 5B). Consistently, SFTSV NSs degraded endogenous SAFA in dose-dependent manner under SFTSV infection (Fig 5C). Next, we assessed the impact of HA-NSs on the half-life of SAFA using cycloheximide treatment. The results demonstrated that the half-life of endogenous SAFA was significantly shortened in HA-NSs overexpressing cells compared with the control (Figs 5D and S3B). The degradation of intracellular proteins includes mainly the autophagy pathway and the ubiquitin-proteasome system. To determine the degradation pathway of SAFA mediated by SFTSV NSs, protease inhibitor MG132 and autophagy inhibitors chloroquine (CQ), and 3-methyladenine (3-MA) were employed. Western blot showed that treatment with CQ or 3-MA greatly restored SAFA levels, whereas no obvious differences in SAFA levels were observed under the treatment of MG132 as compared with the control dimethyl sulfoxide (DMSO) (Fig 5E). Consistently, NSs also degrades SAFA-NLS mutant via autophagy in MEF cells (S3C-S3E Fig). Taken together, these results indicate that SFTSV NSs degrades SAFA in the cytoplasm via autophagy.

### SFTSV NSs mediates the interaction of SAFA and LC3

**Fig 6:**
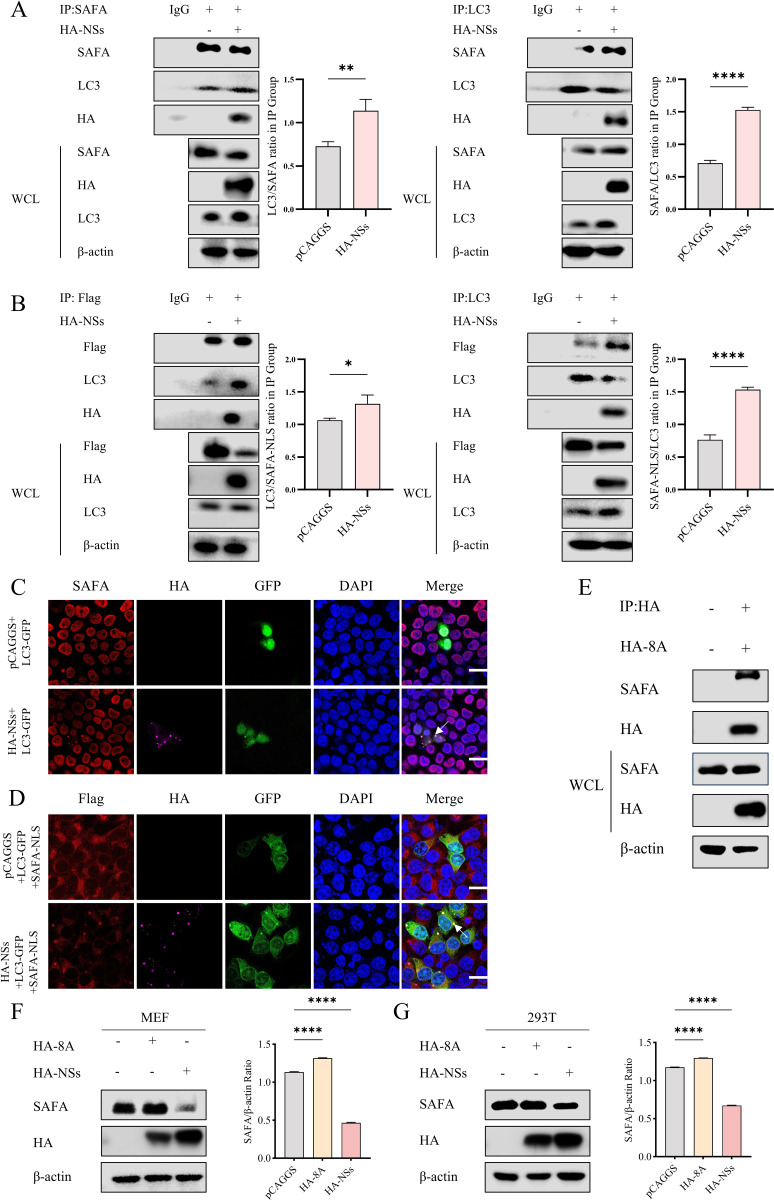
SFTSV NSs mediates the interaction of SAFA and LC3. (A) 293T cells were transfected with HA-NSs plasmid or vector plasmid pCAGGS for 24 h. Then the cell lysates were analyzed with co-IP assays using LC3 or SAFA antibodies. (B) 293T cells were co-transfected with Flag-SAFA-NLS plasmid and HA-NSs plasmid or pCAGGS for 24 h. Then the cell lysates were analyzed with co-IP assays using LC3 or Flag antibodies. (C) 293T cells were transfected with LC3-GFP and pCAGGS or HA-NSs for 24 h. SAFA (red), HA (purple), GFP (green), and DAPI (blue) were analyzed with confocal microscopy. Scale bar: 20 μm. (D) 293T cells were co-transfected with LC3-GFP and Flag-SAFA-NLS with pCAGGS or HA-NSs for 24 h. Flag (red), HA (purple), GFP (green), and DAPI (blue) were analyzed with confocal microscopy. Scale bar: 20 μm. (E) 293T cells were transfected with HA-8A for 24 h. Then the cell lysates were analyzed with co-IP assays using anti-HA antibodies. (F-G) MEF and 293T cells were transfected with pCAGGS, HA-8A, or HA-NSs for 24 h. The protein levels of SAFA were detected with western blot.

SFTSV NSs was demonstrated to induce autophagy and interact with LC3, which is an important autophagy marker. To investigate whether SFTSV NSs induces interaction between SAFA and LC3, HA-NSs and LC3-GFP plasmids were co-transfected into 293T cells. Co-IP assays showed that SFTSV NSs mediated the interaction between SAFA and LC3 (Fig 6A), while the same result was observed in SAFA-NLS and LC3 in the case of HA-NSs transfection (Fig 6B). Confocal microscopy showed that SAFA and SAFA-NLS were clearly co-localized with LC3 upon the transfection of HA-NSs (Fig 6C and 6D). Furthermore, we transfected HA-NSs 8A mutant plasmid that displays a reduced ability in autophagy into 293T cells and MEF cells (S4 Fig). Western blot showed that the 8A mutant still interacted with SAFA (Fig 6E) but degraded significantly less level of SAFA than the wild type HA-NSs (Fig 6F and 6G). Together, these results demonstrate that SFTSV NSs induces autophagy and promotes the interaction between SAFA and LC3 to cause the degradation of SAFA.

### SFTSV NSs degrades SAFA via SQSTM1/p62-dependent selective autophagy

**Fig 7:**
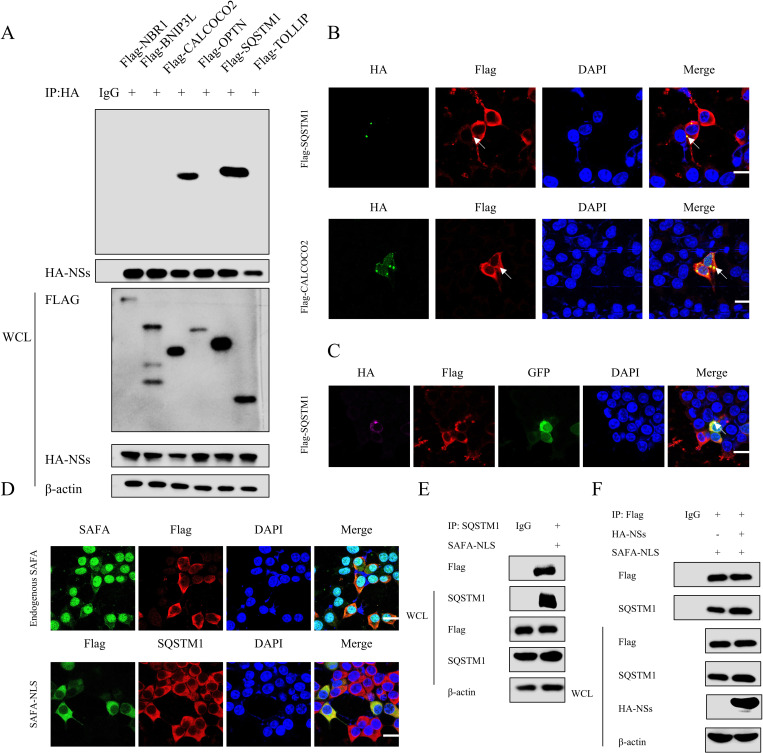
SFTSV NSs promotes the interaction of cytoplasmic SAFA and SQSTM1/p62. (A) 293T cells were co-transfected with HA-NSs and Flag-SQSTM1, Flag-NBR1, Flag-OPTN, Flag-TOLLIP, Flag-CALCOO2 and Flag-BNIP3L for 24 h. The cell lysates were analyzed with co-IP assays using anti-HA antibodies. (B) 293T cells were co-transfected with HA-NSs and Flag- CALCOCO2 or Flag-SQSTM1 plasmids for 24 h. Flag (red), HA (green), and DAPI (blue) were analyzed with confocal microscopy. Scale bar: 20 μm. (C) 293T cells were co-transfected with Flag-SQSTM1, LC3-GFP and HA-NSs for 24 h. Flag (red), HA (purple), GFP (green), and DAPI (blue) were analyzed with confocal microscopy. Scale bar: 20 μm. (D-E) 293T cells were transfected with Flag-SQSTM1 or Flag-SAFA-NLS for 24 h. Flag and SQSTM1 (red), endogenous SAFA and Flag (green), and DAPI (blue) were analyzed with confocal microscopy (D). Scale bar: 20 μm. Then the cell lysates were analyzed with co-IP assays using anti- SQSTM1 antibodies (E). (F) 293T cells were co-transfected with Flag-SAFA-NLS and HA-NSs for 24 h. Then the cell lysates were analyzed with co-IP assays using anti-Flag antibodies.

The autophagic clearance of protein aggregates is a highly selective process that depends on substrate recognition by cargo receptors. To determine which cargo receptor(s) is required for the NSs-mediated autophagic degradation of SAFA, we performed co-IP assays to detect the interactions between NSs and cargo receptors, including SQSTM1/p62, NBR1, OPTN, TOLLIP, CALCOCO2/NDP52 and BNIP3L/NIX. Our results showed that SQSTM1 and CALCOCO2 specifically interacted with NSs (Fig 7A). However, confocal assays showed that SQSTM1 colocalized with NSs in inclusion bodies-like puncta whereas CALCOCO2 did not (Fig 7B). In addition, confocal microscopy showed that NSs colocalized with both SQSTM1 and LC3 (Fig 7C), suggesting SQSTM1 may involve in NSs-induced autophagy. To further examine whether NSs-induced autophagic degradation of SAFA depends on SQSTM1, we analyzed the relationship between SAFA and SQSTM1. Confocal assays demonstrated that only the cytoplasmic SAFA-NLS mutant colocalized with SQSTM1, while the endogenous SAFA did not (Fig 7D). Co-IP assays further confirmed the interaction between SAFA-NLS mutant and SQSTM1 (Fig 7E). To investigate whether SFTSV NSs promotes the interaction between SAFA and SQSTM1, HA-NSs, Flag- SQSTM1 and Flag-SAFA-NLS plasmids were co-transfected into 293T cells and analyzed with co-IP assays. The result showed that SFTSV NSs promoted the binding between SAFA-NLS and SQSTM1 (Fig 7F). These results demonstrated that SQSTM1 may be involved in the autophagic degradation of cytoplasmic SAFA induced by NSs.

## Discussion

Heterogeneous nuclear ribonucleoproteins (HnRNPs) represent a large family of RNA-binding proteins that contribute to multiple aspects of nucleic acid metabolism including alternative splicing, mRNA stabilization, and transcriptional and translational regulation [25]. In recent years, the interaction between viruses and HnRNPs has received increasing attention. Many HnRNPs have been found to be involved in viral infections of DNA viruses and RNA viruses [26,27]. Among them, SAFA plays an antiviral role, restricting the replication of HSV1, VSV, and HIV-1 [20,28]. SAFA has previously been considered a pure nuclear protein. Our previous study showed that SAFA senses RNA viruses in the cytoplasm and plays an important role in anti-RNA virus infection [21]. In this study, SAFA-NLS mutant was overexpressed in SAFA knockout MEF cells as a target protein to explore the function of SAFA as an RNA receptor. These results showed that SAFA-NLS mutant can inhibit SFTSV and further verified that SAFA plays a role in positively upregulating the production of IFN in the cytoplasm to inhibit the replication of RNA viruses. Gupta et al. found that SAFA may be involved in the life cycle and pathogenesis of VSV [29]. Recent studies showed that SAFA negatively regulates the innate immune response against porcine epidemic diarrhea virus and infectious bursal disease virus [30,31]. This means that HnRNP U plays different roles in different hosts or viruses.

Our previous studies showed that SAFA recognized SFTSV NP and RNA and was retained in the cytoplasm by SFTSV NP, and NP promotes the interaction between SAFA and STING to further promote STING-TBK1-dependent antiviral signaling [21]. In this study, we found similar result that NSs interacted with SAFA and retained it in the cytoplasm, and could inhibit SAFA-mediated innate immune responses to achieve immune escape and promote viral proliferation. Our study indicated that the effects of NP and NSs on SAFA were different though they were both reported to participate in immune escape through autophagy. Compared with NP, NSs is more conservative as immune escape molecule. In view of that SFTSV NP and NSs can interact with SAFA and mediate its nuclear-cytoplasmic translocation, it is not ruled out that other components of SFTSV could also interact with SAFA. Therefore, further study is needed to explore the relationship between SAFA and the components of SFTSV. In addition, it will be interesting to explore the relationship within the components of SFTSV.

As a traditional cellular defense mechanism, autophagy can degrade invading virus particles, control viral replication, and activate immune responses [32]. However, more and more studies related to autophagy and viral immune evasion have been reported [33,34]. Previous studies reported that SFTSV NSs act by sequestering multiple host molecules into viral inclusion bodies [35,36]. Previous study showed that NSs induced autophagy to recruit TBK1, TRIM25 and other antiviral immune proteins into the autophagic flux for degradation [18]. In this study, we demonstrated that SFTSV NSs promotes the interaction of LC3 and SAFA inducing autophagy to degrades SAFA and thereby to escape the antiviral immune response of SAFA. This study further strengthens the idea that NSs evade host innate immune responses through autophagy. It is worth noting that in this study, we found that the NSs can inhibit the phosphorylation of TBK1, p65 and other molecules induced by SAFA. A previous study reported that SFTSV NSs can directly degrade TBK1 and p65 through autophagy and thereby inhibit the production of IFN [18]. We found that deletion of SAFA did not affect the degradation of TBK1 and p65 by NSs (S5 Fig). Our findings revealed that the two pathways of NSs inhibiting the phosphorylation of TBK1 and p65 by degrading SAFA and NSs directly degradation of TBK1 and p65 to reduce their phosphorylation were independent of each other. It indicated that NSs utilize autophagy through multiple strategies for immune evasion.

Previously, we found that mutating the 8A motif of NSs could eliminate the immune evasion induced by NSs through autophagy. Similarly, in this study, we found that the 8A mutant can interact with SAFA but cannot degrade SAFA, which indicates that although the 8A sequence of mutant NSs cannot completely eliminate autophagy induction, it loses the ability to recruit SAFA into autophagic flux for degradation. Our study further confirmed the importance of the 8A motif of NSs in its induction of autophagy and immune evasion indicating a potential developing anti-SFTSV therapeutic strategies by targeting the NSs-LC3 interaction.

Our previous studies have shown that SFTSV infection may promote viral replication or immune evasion by modulating the host cell’s autophagy pathway. Additionally, we found that SFTSV NP degrades MAVs through TUFM-mediated autophagy. In this study, we discovered that SFTSV NSs interacts with the autophagy receptor SQSTM1, which plays a crucial role in selective autophagy. The mutated cytoplasmic SAFA interacts with SQSTM1, suggesting that NSs promote SQSTM1-mediated SAFA degradation by facilitating SAFA nucleocytoplasmic translocation. Although this study reveals that NSs promote SAFA nucleocytoplasmic translocation and enhance the interaction between SAFA, SQSTM1, and LC3, further study needs to be explored. Notably, we also found that, in addition to SQSTM1, cargo receptor CALCOO2 also interacted with NSs which indicated other strategy of NSs to escape immune response. Together, these findings reveal the complexity of the mechanisms employed by viruses for the autophagic degradation of host proteins.

**Fig 8:**
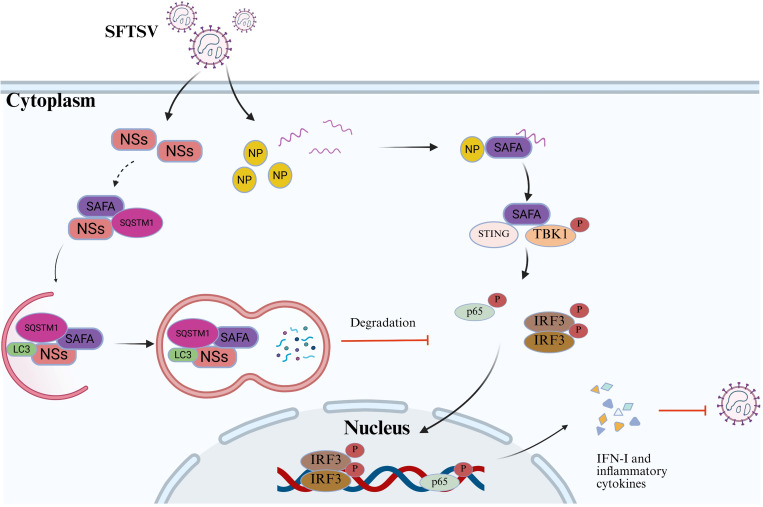
Schematic model illustrating the interaction between SFTSV NSs and SAFA. SFTSV NSs interacts with SAFA and retains it in cytoplasm. SFTSV NSs induces SQSTM1-dependent autophagy and promotes the interaction between SAFA and LC3, leading to SAFA degradation, and thereby suppressing SAFA-mediated innate immune response. The model was created in BioRender. yu, t. (2025) https://BioRender.com/vgmkiln.

In conclusion, our study shows that the interaction between SFTSV NSs and the novel RNA sensor SAFA retains SAFA in the cytoplasm and mediates autophagic degradation of SAFA through interaction with LC3 and SQSTM1, thereby inhibiting SAFA-mediated innate immune responses and promoting viral replication (Fig 8). These findings reveal a new strategy of SFTSV NSs to mediate immune evasion and promote viral replication.

## Materials and methods

### Cells and viruses

293T, MEF, and Vero cells were cultured in Dulbecco’s modified Eagle’s medium (DMEM) (BBI, E600003), supplemented with 10% fetal bovine serum (BBI, E600001) and 1% penicillin-streptomycin-G (Beyotime, C0223) at 37°C with 5% CO_2_. SAFA ^-/-^ MEF cells was constructed with CRISPR-Cas9 system as previously reported [21]. SFTSV (strain JS2011-013-1) was propagated in Vero cells and stored at -80˚C for use. VSV-GFP was kindly provided by Professor Bo Zhong (Wuhan University, Wuhan, China). Nuclear and cytoplasmic protein extraction kit (Solarbio, R005) was utilized in 293T and MEF cells for separation of nucleus and cytoplasm after Flag-SFTSV NSs transfection for 24 h.

### Antibodies and reagents

The primary antibodies, chemical reagents, and their manufactures used in the study are listed below: HnRNP U (Santa Cruz Biotechnology, sc-32315), phospho-TBK1(ABconal, AP1026), IRF3 (ABconal, A1118), Phospho-p65 (Cell Signaling Technology, 93H1), TBK1 (Cell Signaling Technology, 3504S), LC3B (Cell Signaling Technology, 3868S), p65 (Cell Signaling Technology, D14E12), Phospho-IRF3 (Proteintech, 29528–1-AP), p65 (Proteintech, 10745–1-AP), SQSMT1 (Proteintech, CL594–6618), Flag-tag (Proteintech, 20543–1-AP, 66008–1-AP), HA-tag rabbit (Proteintech, 14599–1-AP, 66006–1-AP), GFP-tag (Proteintech, 66002–1-Ig), His-tag (Proteintech, 66005–1-AP) and HRP-conjugated goat anti-human secondary antibody (Proteintech, bs-0297G-HRP), ACTB (Abbikine, ABL1010), HRP-conjugated goat anti-mouse secondary antibody (Abbikine, A21010), goat anti-rabbit secondary antibody (Abbikine, A21020), Fluorescence-labeled secondary antibodies, including Alexa Fluor 488 goat anti-rabbit IgG IgM (H + L) (Thermo Fisher, A11008), Alexa Fluor 594 goat anti-mouse IgG (Thermo Fisher, A11005), Alexa Fluor 647 goat anti-rabbit IgG (H + L) (Thermo Fisher, A21245. Alexa Fluor 488 goat anti-human IgG, IgM (H + L) (Solarbio, K1001G-AF488). Poly(I:C) (LMW) (tlrl-picwlv) (InvivoGen, HY-107202), Chloroquine (CQ) (Sigma Aldrich, C6628), 3-Methyladenine (3-MA) (Beyotime, HY-19312), Dimethyl sulphoxide (DMSO) (Solarbio, D8371). Primary antibodies specific for SFTSV NP were produced in our laboratory.

### Plasmids and DNA transfection

PCDNA3.1 (+)-3 × Flag-U1 and pCDNA3.1 (+)-3 × Flag-U2, pCDNA3.1 (+)-3 × Flag-U3, pCDNA3.1 (+)-3 × Flag-U4, pCDNA3.1 (+)-3 × Flag-SPRY, pCDNA3.1 (+)-3 × Flag-RGG and HA-8A, and PCDNA3.1 (+)-3 × Flag-SAFA-NLS were constructed by Tsingke Biotechnology. Flag-SQSTM1, Flag-NBR1, Flag-OPTN, Flag-TOLLIP, Flag-CALCOO2 and Flag-BNIP3L were kindly provided by Professor Xing Liu (Nanjing Agricultural University, Nanjing, China). Hieff Trans Liposomal Transfection Reagent (40802ES02) (YEASEN) was used as transfection reagent.

### Western blot analysis

Cells were lysed on ice with 1 × SDS loading buffer (Servicebio, G2013), briefly ultrasonicated, and then heated at 95°C for 10 min. Protein samples were separated using SDS-polyacrylamide gel electrophoresis and subsequently transferred to polyvinylidene difluoride membranes (Millipore, IPVH00010). The membranes were blocked with 5% non-fat milk in 1 × Tris-buffered saline (TBS) (Solarbio, T1080) and 0.05% Tween 20 (Solarbio, T8220) for 1 h and incubated with corresponding primary antibodies overnight at 4°C, followed by HRP-conjugated secondary antibodies for 1 h. The protein levels were detected using hypersensitive ECL Chemiluminescence Detection Kit (Yeasen Biotechnology, 36208ES76) in ChemiDoc Touch Imaging System (Bio-Rad) and analyzed with ImageLab software.

### Co-immunoprecipitation assay

Cells were lysed on ice with NP-40 lysis buffer (Beyotime, ST2045), briefly ultrasonicated, and then centrifuged at 12,000 × g for 5 min at 4°C. The lysates were incubated with the corresponding antibodies and control IgG antibodies overnight at 4°C and then incubated with protein A/G agarose (Beyotime, P2055) for 2–3 h at 4°C. The mixture was washed with PBS five times 5 min each by centrifugation at 12,000 × g at 4°C. The beads were resuspended with 1 × SDS loading buffer for western blot analysis.

### RNA extraction and RT-qPCR

Total RNA was extracted using TRIzol Reagent (Servicebio, G3013) and then transcribed into cDNA with NovoScript Plus All-in-one 1st Strand cDNA Synthesis SuperMix (gDNA Purge) (E047-01B) (Novoprotein) according to the manufacturer’s instructions. RT-qPCR was performed with specific primers and NovoStart SYBR qPCR SuperMix Plus (Novoprotein, E096-01B) using a CFX96 touch system (BIO-RAD). Relative mRNA expression levels were calculated by the 2 − ΔΔCt method, normalizing with ACTB. The primers used were listed in S1 Table.

### Immunofluorescence and confocal microscopy

After transfected with indicated plasmids for 24 h, cells were fixed with 4% paraformaldehyde for 20 min, permeabilized with 0.2% Triton X-100 (Solarbio, T8200), blocked with 2% bovine serum albumin (Solarbio, A8020) for 1 h, and then incubated with indicated primary antibodies at 4°C overnight, followed by fluorescence labeled secondary antibodies for 1 h. Nuclei were stained with DAPI (Beyotime, C1005) for 10 min. Cells were observed using Leica sp8 confocal laser microscope (Zeiss LSM880). All images analyses were performed using the software Leica Application Suite X.

### RNA immunoprecipitation (RIP) assay

RNA immunoprecipitation was performed using the RNA Immunoprecipitation Kit (BersinBio, Guangzhou, China). MEF cells were transfected with Flag-SAFA-NLS plasmid, and then infected with SFTSV (MOI = 5) for 24 h. After infection, the cells were harvested and lysed using the provided lysis buffer to extract the RNA-protein complexes. The cell lysate was incubated overnight at 4˚C with magnetic protein A/G beads conjugated with anti-Flag antibody or IgG as a negative control. The immunoprecipitated SAFA and RNA was extracted and analyzed with western blot and nPCR, respectively. The primers used were listed in S2 Table.

### Median tissue culture infectious dose (TCID_50_)

The collected viruses were serially diluted 10-fold in a suitable maintenance medium. Vero cells seeded in a 96-well plate were infected with the diluted viral stocks, using 8–16 wells per dilution gradient. The plates were then incubated at 37°C with 5% CO₂ for 24 hours to allow viral infection and replication. After 24 h incubation, the monolayers were fixed with 4% paraformaldehyde for 30 min and permeabilized with 0.2% Triton-100 for 10–15 min. After washing with PBS and blocking with 1% BSA, the cells were incubated with primary antibodies specific to the SFTSV NP at 4^o^C overnight; followed by incubating Goat Anti-Human IgG/FITC secondary antibody (Proteintech, Wuhan, China) for 30 min in the dark. The fluorescence was observed under a fluorescence microscope. The virus titer was calculated by Reed Muench method.

### Statistical analysis

Statistical analyses were performed using Student’s t-test or one-way ANOVA with GraphPad Prism 9. P-value equal to or lower than 0.05 was considered statistically significant (n = 3; *p < 0.05, **p < 0.01, ***p < 0.001, ****p < 0.0001 and ns, no significance).

## Supporting information

S1 Fig SAFA-NLS mutant localized in cytoplasm. **(A)** Knockout of SAFA in MEF cells was identified with western blot. **(B)** *SAFA*^-/-^ MEF cells were transfected with Flag-SAFA-NLS mutant plasmid for 24 h. Cells were then collected at 24 h for nuclear and cytosolic fractionation assay. Lamin A and Rab5 were nuclear and cytoplasmic index proteins, respectively. **(C)** *SAFA*^-/-^ MEF cells were transfected with Flag-SAFA-NLS mutant plasmid or pCDNA3.1 for 24 h. SAFA-NLS (red), and DAPI (blue) were analyzed with confocal microscopy. Scale bar: 20 μm. **(D)** *SAFA*^-/-^ MEF cells were transfected with Flag-SAFA-NLS mutant plasmid for 24 h. The interaction between the S, M and L segment of SFTSV RNA and SAFA-NLS was detected by RIP.
(TIF)

S2 Fig SAFA-NLS mutant directly interacts with SFTSV NSs. **(A-B)** 293T cells were co-transfected with Flag-SAFA-NLS plasmid and pCAGGS or HA-NSs for 24 h **(A)** 293T cells were infected with SFTSV for 2 h and then co-transfected with Flag-SAFA-NLS plasmid and pCAGGS or HA-NSs for 24 h for simulating the infection status **(B)**. The interaction between endogenous SAFA and SAFA-NLS and NSs was analyzed with co-IP assay using IgG as control. **(C)** 293T cells were co-transfected with Flag-SAFA-NLS plasmid and pCAGGS or HA-NSs for 24 h and then with or without SFTSV (MOI = 5) infection for 24 h. HA (green), Flag (red), and DAPI (blue) were analyzed with confocal microscopy. Nuclei were stained with DAPI (blue). Scale bar: 20 μm.
(TIF)

S3 Fig NSs degrades SAFA-NLS via autophagy. **(A)** MEF and 293T cells were transfected with the increasing amount of HA-NSs plasmid for 24 h. The protein levels of SAFA were detected with RT-qPCR. **(B)** Western blot data (Fig 5D) were semi-quantified and normalized against β-actin protein loading control. **(C)** MEF cells were co-transfected with Flag-SAFA-NLS mutant and different doses of HA-NSs plasmid for 24 h. The protein levels of SAFA-NLS were detected using western blot with anti-Flag antibody. **(B)** MEF cells were transfected with Flag SAFA-NLS mutant plasmid and HA-NSs plasmid were cultivated for different time periods. The protein levels of SAFA-NLS were detected with western blot using anti-Flag antibody. **(E)** MEF cells were transfected with Flag-SAFA-NLS plasmid and HA-NSs for 24 h, and then the cells were treated with DMSO, CQ (5μM), 3-MA (1mM), and MG132 (5μM). The protein levels of SAFA-NLS were detected with western blot using anti-Flag antibody.
(TIF)

S4 Fig HA-8A mutant of NSs displays a reduced ability in autophagy. **(A-B)** 293T cells were transfected with pCAGGS, HA-8A, or HA-NSs plasmid for 24h, and LC3 (green), HA (red), and DAPI (blue) were analyzed with confocal microscopy **(A)**. LC3 protein levels was detected with western blot **(B)**.
(TIF)

S5 Fig SAFA does not affect the degradation of TBK1 and p65 by NSs. **(A)** WT or *SAFA*^-/-^ MEF cells were transfected with HA-NSs plasmid for 24 h. The protein levels of TBK1 and p65 were analyzed with western blot.
(TIF)

S1 Table Primers used for RT-qPCR. (DOCX)

S2 Table Primers used for nPCR. (DOCX)
