# Supplementary figures and images for: SFTSV NSs degrades SAFA via autophagy to suppress SAFA-dependent antiviral response
Source: PLoS Pathog. 2025 Jun 3;21(6):e1013201. doi: 10.1371/journal.ppat.1013201 (PMC12132933; doi:10.1371/journal.ppat.1013201)

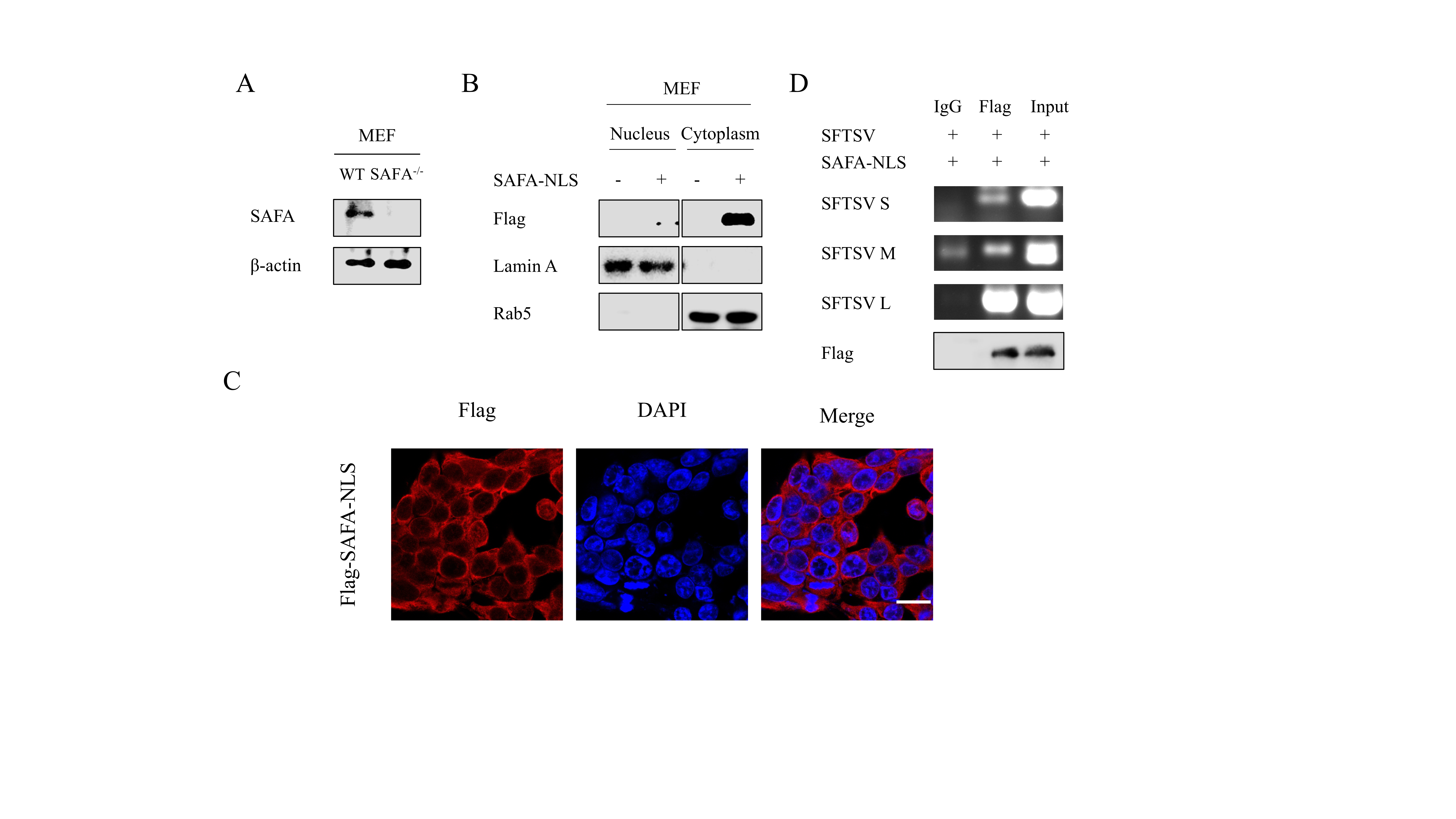

Supplement: S1 Fig — (A) Knockout of SAFA in MEF cells was identified with western blot. (B) SAFA-/- MEF cells were transfected with Flag-SAFA-NLS mutant plasmid for 24 h. Cells were then collected at 24 h for nuclear and cytosolic fractionation assay. Lamin A and Rab5 were nuclear and cytoplasmic index proteins, respectively. (C) SAFA-/- MEF cells were transfected with Flag-SAFA-NLS mutant plasmid or pCDNA3.1 for 24 h. SAFA-NLS (red), and DAPI (blue) were analyzed with confocal microscopy. Scale bar: 20 μm. (D) SAFA-/- MEF cells were transfected with Flag-SAFA-NLS mutant plasmid for 24 h. The interaction between the S, M and L segment of SFTSV RNA and SAFA-NLS was detected by RIP. (TIF) [file ppat.1013201.s001.tif]

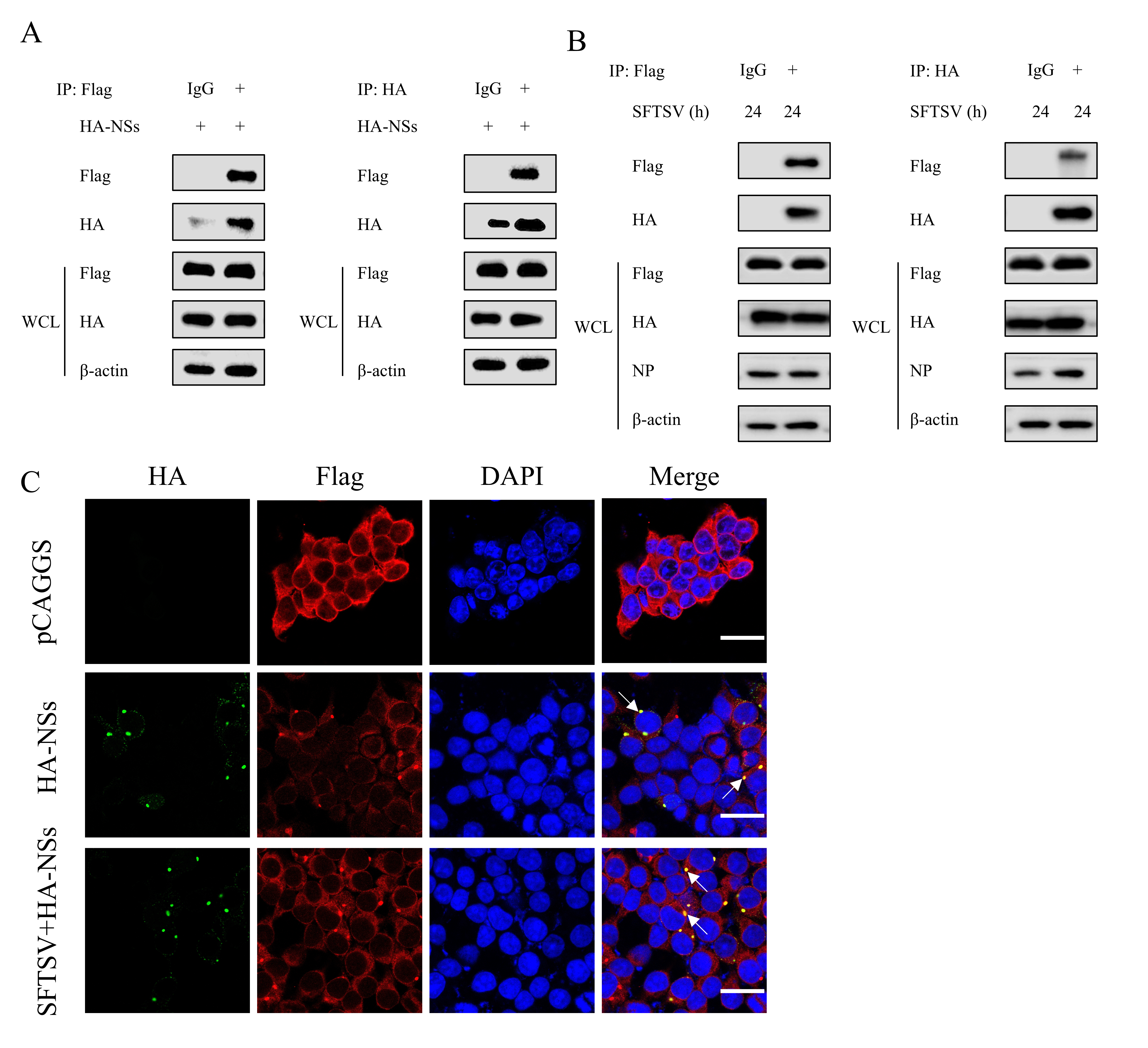

Supplement: S2 Fig — (A-B) 293T cells were co-transfected with Flag-SAFA-NLS plasmid and pCAGGS or HA-NSs for 24 h (A) 293T cells were infected with SFTSV for 2 h and then co-transfected with Flag-SAFA-NLS plasmid and pCAGGS or HA-NSs for 24 h for simulating the infection status (B). The interaction between endogenous SAFA and SAFA-NLS and NSs was analyzed with co-IP assay using IgG as control. (C) 293T cells were co-transfected with Flag-SAFA-NLS plasmid and pCAGGS or HA-NSs for 24 h and then with or without SFTSV (MOI = 5) infection for 24 h. HA (green), Flag (red), and DAPI (blue) were analyzed with confocal microscopy. Nuclei were stained with DAPI (blue). Scale bar: 20 μm. (TIF) [file ppat.1013201.s002.tif]

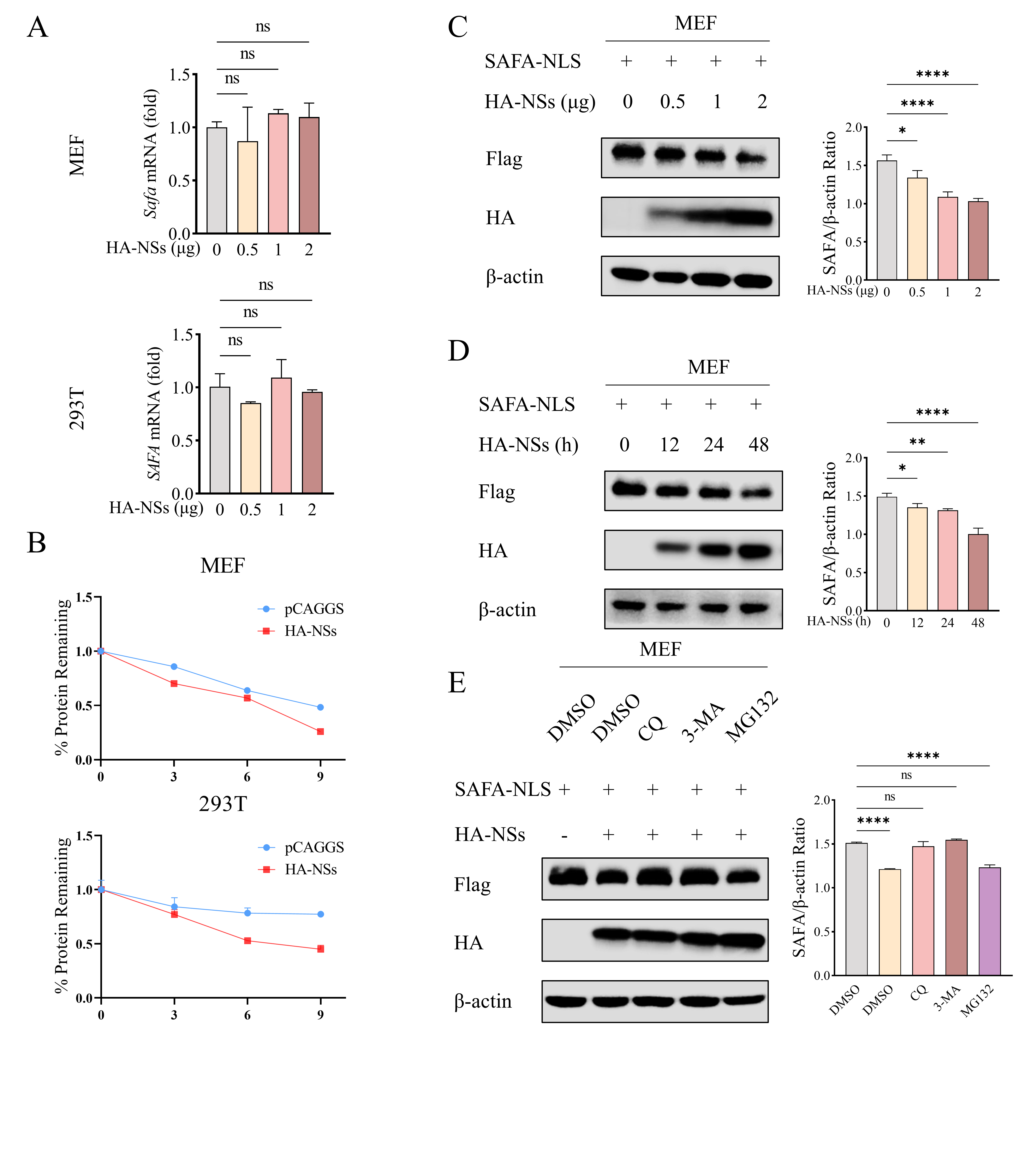

Supplement: S3 Fig — (A) MEF and 293T cells were transfected with the increasing amount of HA-NSs plasmid for 24 h. The protein levels of SAFA were detected with RT-qPCR. (B) Western blot data (Fig 5D) were semi-quantified and normalized against β-actin protein loading control. (C) MEF cells were co-transfected with Flag-SAFA-NLS mutant and different doses of HA-NSs plasmid for 24 h. The protein levels of SAFA-NLS were detected using western blot with anti-Flag antibody. (B) MEF cells were transfected with Flag SAFA-NLS mutant plasmid and HA-NSs plasmid were cultivated for different time periods. The protein levels of SAFA-NLS were detected with western blot using anti-Flag antibody. (E) MEF cells were transfected with Flag-SAFA-NLS plasmid and HA-NSs for 24 h, and then the cells were treated with DMSO, CQ (5μM), 3-MA (1mM), and MG132 (5μM). The protein levels of SAFA-NLS were detected with western blot using anti-Flag antibody. (TIF) [file ppat.1013201.s003.tif]

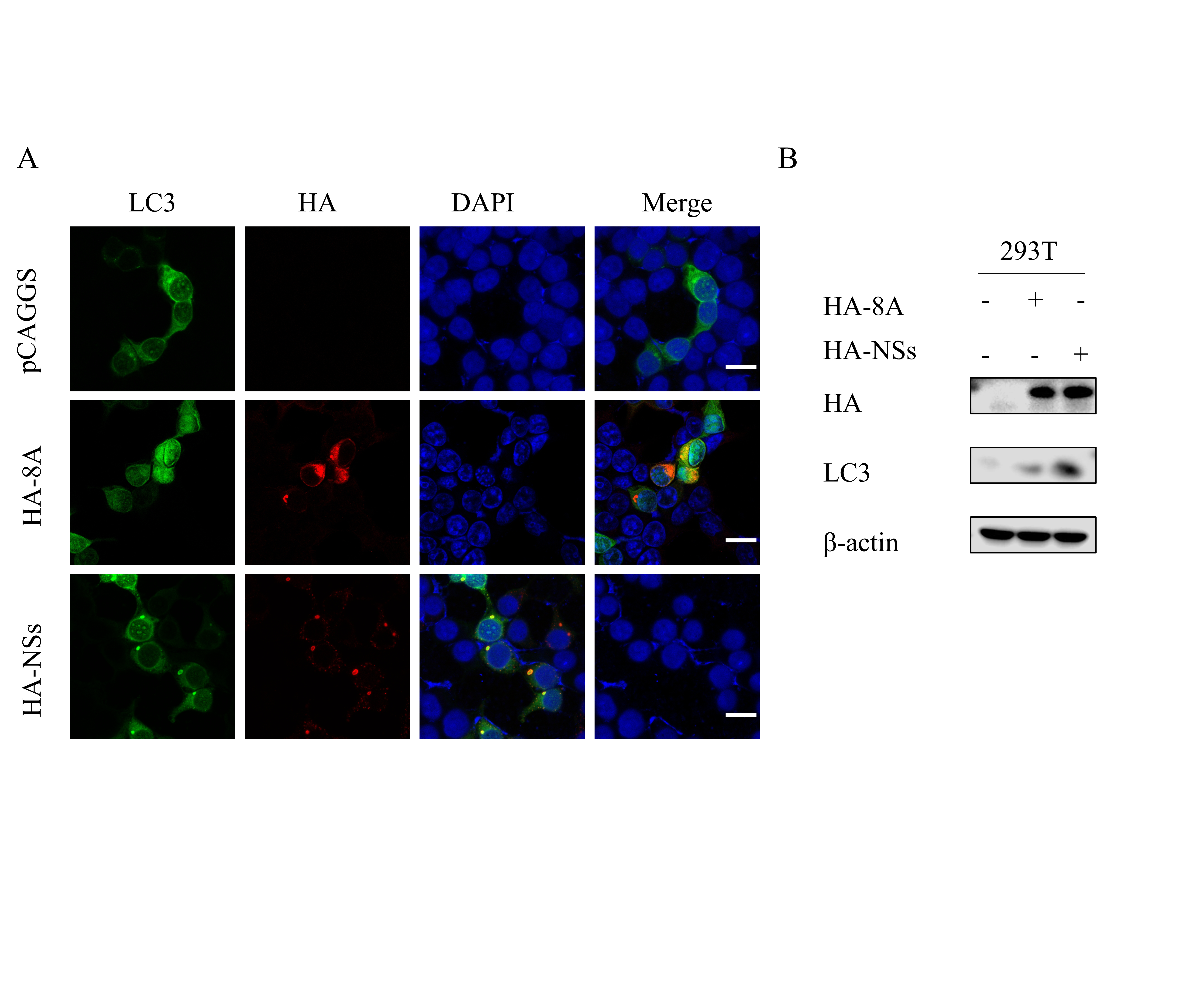

Supplement: S4 Fig — (A-B) 293T cells were transfected with pCAGGS, HA-8A, or HA-NSs plasmid for 24h, and LC3 (green), HA (red), and DAPI (blue) were analyzed with confocal microscopy (A). LC3 protein levels was detected with western blot (B). (TIF) [file ppat.1013201.s004.tif]

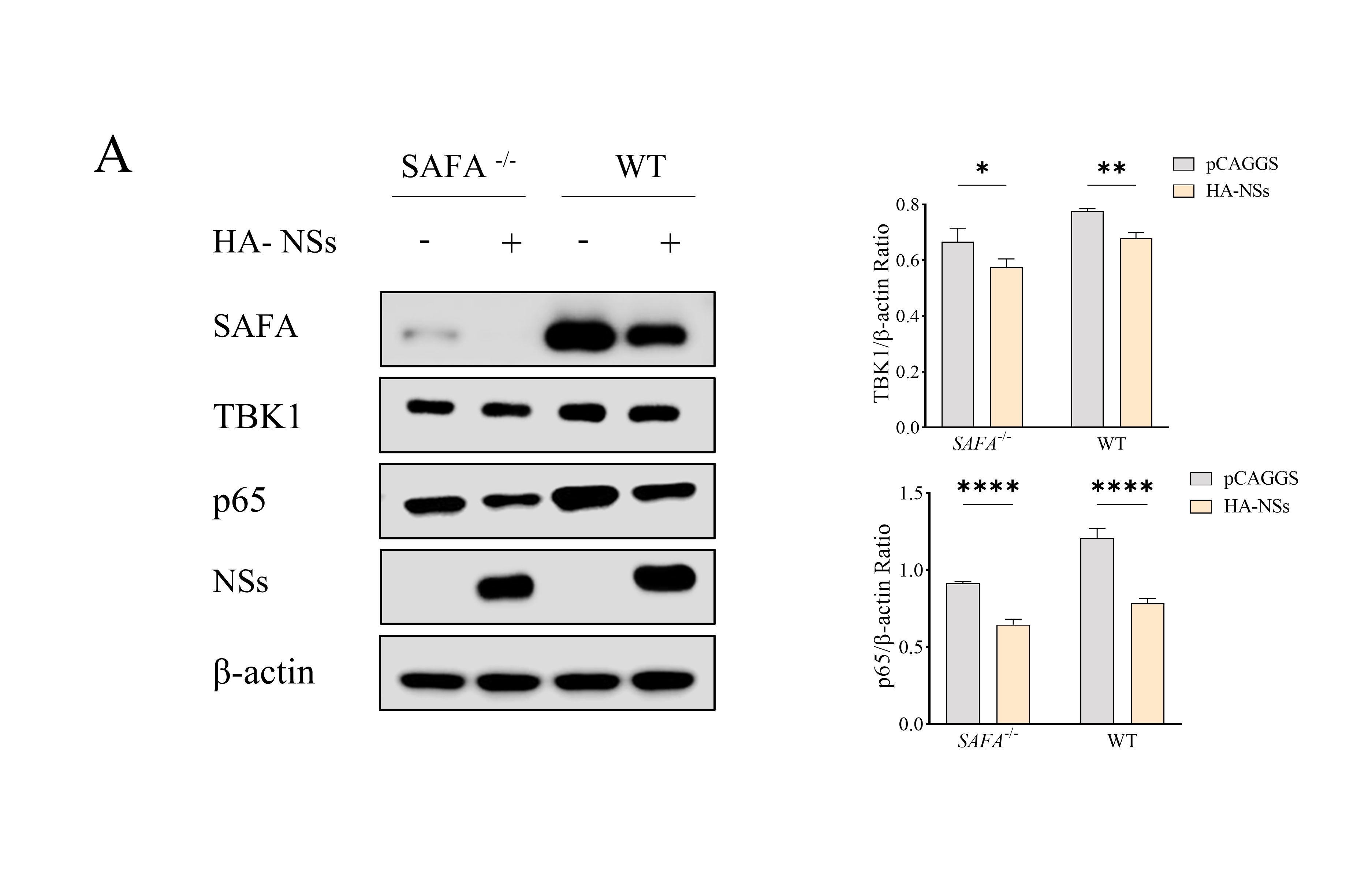

Supplement: S5 Fig — (A) WT or SAFA-/- MEF cells were transfected with HA-NSs plasmid for 24 h. The protein levels of TBK1 and p65 were analyzed with western blot. (TIF) [file ppat.1013201.s005.tif]
