# Supplementary material for: SFTSV NSs degrades SAFA via autophagy to suppress SAFA-dependent antiviral response
Source: PLoS Pathog. 2025 Jun 3;21(6):e1013201. doi: 10.1371/journal.ppat.1013201 (PMC12132933; doi:10.1371/journal.ppat.1013201)
Supplement: S1 Table — (DOCX) [file ppat.1013201.s006.docx]

**S1 Table. Primers used for RT-qPCR.**

| Primer name | Forward primer sequence | Reverse primer sequence |
| --- | --- | --- |
| Humo SAFA | AGGAAGTTCTTGCTGGACGG | GGCCCCTTTGGTCCTCTAAC |
| Murine SAFA | AAGGAGGAGCTCAAGAAGCG | CTCCTGCCTCGTTGTCCAG |
| Murine IFNβ | TCCGAGCAGAGATCTTCAGGAA | TGCAACCACCACTCATTCTGAG |
| Murine IL-1β | ACTGTTTCTAATGCCTTCCC | ATGGTTTCTTGTGACCCTGA |
| Murine CXCL10 | CCTGCCCACGTGTTGAGAT | GAGTCACAGACCCGTCCCTA |
| Mouse IFIT1 | GTCCGGTTAAATCCAGAAGATCC | GCTTTGTCTACGCGATGTTTCC |
| Murine β-actin | AGAGGGAAATCGTGCGTGAC | CAATAGTGATGACCTGGCCGT |
| SFTSV NP | TGTCAGAGTGGTCCAGGATT | ACCTGTCTCCTTCAGCTTCT |
