# Supplementary material for: SFTSV NSs degrades SAFA via autophagy to suppress SAFA-dependent antiviral response
Source: PLoS Pathog. 2025 Jun 3;21(6):e1013201. doi: 10.1371/journal.ppat.1013201 (PMC12132933; doi:10.1371/journal.ppat.1013201)
Supplement: S2 Table — (DOCX) [file ppat.1013201.s007.docx]

**S2 Table. Primers used for nPCR.**

| Primer name | Forward primer sequence | Reverse primer sequence |
| --- | --- | --- |
| Step 1 |  |  |
| S segment | CAGCCAGTTTACCCGAACAT | GAAAGACGCAAAGGAGT |
| M segment | TCTGCAGTTCAGACTCAGGGA | GACGTGTATTGCTGTTTTCCCC |
| L segment | AATGATGCCAAGAAGTGGAAT | ATGTAAGCATAGTCCTAGAAGC |
| Step 2 |  |  |
| S segment | TGGCTCCGCGCATCTTCACA | AGAGTGGTCCAGGATTGCTGTGG |
| M segment | TGTTGCTTGTCAGCCTATGAC | CAACCAATGATCCTGAGTGGA |
| L segment | CCACAGATTCATTTGGGCT | ATCATGATCGCTGAGTCGTC |
